# The relationship between quantitative epicardial adipose tissue based on CT and coronary artery disease: A protocol for systematic review and meta-analysis

**DOI:** 10.1097/MD.0000000000023729

**Published:** 2020-12-18

**Authors:** Baohua Wu, Zhuanqin Ren, Zhengang Du, Lei Zhang, Bin Hou

**Affiliations:** a Baoji Central Hospital, Baoji 721000, Shaanxi Province; b Gansu gem flower hospital, Lanzhou 730060, Gansu Province; c Baoji hi-tech people's hospital, Baoji 721013, Shaanxi Province, China.

**Keywords:** coronary artery disease, CT, epicardial adipose tissue, radiography, scheme, systematic review

## Abstract

**Background:**

Epicardial adipose tissue (EAT) is a kind of visceral adipose tissue with close proximity to coronary artery and myocardium, which can secrete cell factor, and influence the physiological function and pathophysiological process of myocardium and coronary artery. Clinical imaging diagnosis showed that the volume and thickness of EAT exists a certain relevance with coronary artery disease, but it lacked evidence of evidence-based medicine. The research on the implementation of this program will systematically evaluate the relationship of computed tomography (CT) quantitative EAT and coronary artery disease.

**Method:**

The English databases (Embase, PubMed, the Cochrane Library, Web of Science) and Chinese database (CNKI, Wanfang, China biomedical database, VIP) of computer retrieval has collected the case control clinical study of relationship between EAT and coronary artery disease from the establishment of the database to October 2020, which was conducted extraction and quality evaluation by 2 researchers independently for data included in the study, and was conducted Meta-analysis for the included literature by adopting RevMan5.3 software.

**Result:**

The research evaluated the correlation between EAT and coronary artery disease through the EAT thickness, EAT volume, and other indexes.

**Conclusion:**

The research has provided reliable evidence-based evidence for the correlation between CT EAT quantification and coronary artery disease.

**Ethics and dissemination:**

We will not publish private information from individuals. This kind of systematic review does not involve harming the rights of participants. No ethical approval was required. The results can be published in peer-reviewed journals or at relevant conferences.

**OSF Registration number:**

DOI 10.17605/OSF.IO/DVQNE

## 1 Introduction

Epicardial adipose tissue is the adipose tissue between myocardium and cardiac valve layer, mainly depositing in atrial sulcus and interventricular sulcus, and covering most of the blood vessels in the heart, whose lesion may cover the whole heart.^[1]^ Epicardial adipose tissue is directly supplied with blood by coronary artery closely contacting with myocardial tissue to provide anatomical basis for the effect of epicardial adipose tissue on heart. From the physiological point of view, epicardial adipose tissue can have the function of providing energy, absorbing metabolites, and maintaining heart temperature with mechanical protection.^[2]^ From the perspective of pathophysiology, epicardial adipose tissue can secrete a large number of anti-inflammatory and pro-inflammatory adipokines.^[3]^ A large number of adipokines may increase the risk of cardiac metabolism under the condition of pathological changes, so as to induce the cardiovascular disease.^[4]^

At present, imaging method for detecting epicardial adipose tissue mainly includes ultrasound, computed tomography (CT), magnetic resonance imaging (MRI), CT scan, which are common, intuitive, and economical, with easy operation, which is less affected by human factors and has great advantages in clinical diagnosis.^[5]^

At present, many clinical studies have shown that there is a certain correlation between CT quantitative epicardial adipose tissue and coronary artery disease.^[6,7]^ Moreover, CT measurement results are accurate and error is small, but the number of clinical trials is small with a certain methodological differences, and the relationship between epicardial adipose tissue and coronary artery disease is still controversial. Therefore, in this study, we conducted a meta-analysis to explore the relationship between epicardial adipose tissue and coronary artery disease, which provides new evidence-based evidence for the clinical prediction or diagnosis of coronary artery disease.

## 2 Method

### 2.1 Protocol register

This protocol of systematic review and meta-analysis has been drafted under the guidance of the preferred reporting items for systematic reviews and meta-analyses protocols (PRISMA-P). Moreover, it has been registered on open science framework (OSF) on November 12, 2020. (Registration number: DOI 10.17605/OSF.IO/DVQNE).

### 2.2 Ethics

The literature study was not related to the personal data included in the study, so the approval of the ethics committee is not required.

### 2.3 Eligibility criteria

#### 2.3.1 Types of studies

We will collect all case--control studies that measure epicardial adipose tissue with CT and explore its association with coronary artery disease, without the limitation of publication status, region, and time, but the language is limited to Chinese and English.

#### 2.3.2 Research objects

A group of suspected patients for imaging diagnosis of coronary artery disease is selected without the limitation of nationality, race, age, gender, course of disease of samples induced into the study, and other special complications.

#### 2.3.3 Interventions

After the coronary angiography or coronary imaging was adopted to judge the degree of coronary artery stenosis, they were divided into coronary artery stenosis group (coronary artery stenosis ≥50%) and noncoronary artery stenosis group (coronary artery stenosis <50%), and CT was used to scan and detect the epicardial adipose tissue of 2 groups of patients.

#### 2.3.4 Outcome indicators

(1) Main outcome: Epicardial adipose tissue volume
(2) Secondary outcome: Epicardial tissue thickness, epicardial adipose tissue threshold

### 2.4 Exclusion criteria

(1) Repeat the published research and the conduct case duplication study;
(2) Research with incomplete data or obvious errors;
(3) Research with incomplete data or obvious errors; studies published with abstracts, expert experience summaries, conference papers, and case reports that cannot obtain the original data;
(4) Adopt other imaging means for detection without adopting the quantitative study of CT;
(5) The subjects have other factors for coronary heart disease, such as congenital heart disease, diabetes, valve disease, and so on.

### 2.5 Search strategy

In 4 English databases, PubMed, Cochrane Library, EMBASE, web of science, and 4 Chinese databases, CNKI, Wanfang, VIP, and China biomedical database, there are 2 researchers who collected all the literatures about the relationship between epicardial adipose tissue and coronary artery disease by CT according to retrieval strategy, and the retrieval time is from the establishment of the database to October 2020. PubMed was taken as the example, and the specific retrieval strategies are summarized in Table 1.

**Table 1:** Search strategy in PubMed database.

Number	Search terms
#1	Coronary artery disease[MeSH]
#2	Coronary artery disease[Title/Abstract]
#3	Coronary arteriosclerosis[Title/Abstract]
#4	Coronary Atherosclerosis[Title/Abstract]
#5	#1 OR #2 OR #3 OR #4
#6	Epicardial fat[Title/Abstract]
#7	Epicardial adipose tissue[Title/Abstract]
#8	Subepicardial fat[Title/Abstract]
#9	Subepicardial adipose tissue[Title/Abstract]
#10	#6 OR #7 OR #8 OR #9
#11	Tomography, X-Ray Computed[Mesh]
#12	Tomography, X-Ray Computed[Title/Abstract]
#13	X-Ray Computed Tomography[Title/Abstract]
#14	X-Ray Computerized Axial Tomography[Title/Abstract]
#15	CT Scan, X Ray[Title/Abstract]
#16	CAT Scan, X Ray[Title/Abstract]
#17	#11 OR #12 OR #13 OR #14 OR #15 OR #16
#18	#5 And #10 And #17

### 2.6 Data screening and extraction

**Figure 1:**
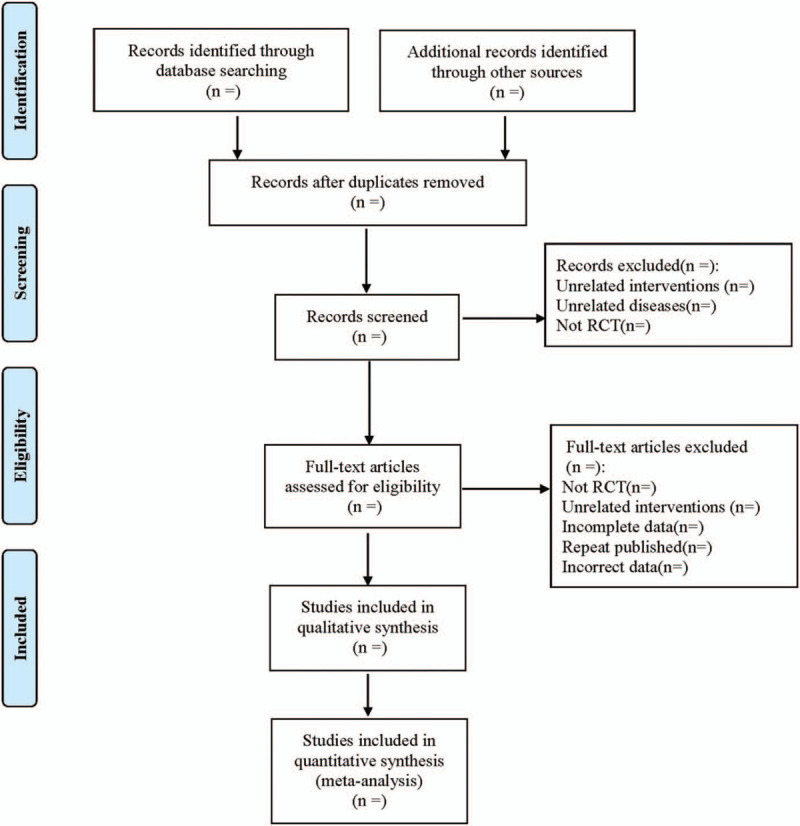
The process of literature filtering.

According to the PRISMA flowchart, the documents were screened independently according to the inclusion and exclusion criteria mentioned above, before 2 researchers used EndNote X7 document management software to conduct mutual inspection. Two researchers used Excel 2013 to extract the first author, year of publication, sample size, number of case--control cases and measurement methods, and results of epicardial adipose tissue, and measured the fat threshold and other information of epicardial adipose tissue volume according to the inclusion and exclusion criteria independent reading of literature, and when the extracted information is inconsistent, it is decided by the third party. The literature screening process is shown in Figure 1.

### 2.7 Literature quality evaluation

Two researchers respectively evaluated the literature quality according to Newcastle--Ottawa Scale (NOS) table, including the selection of research objects, comparability between groups, measurement of exposure factors, and so on. If there is a disagreement, they will discuss it. If no agreement can be reached, a decision will be made after consultation with the third-party researchers.

### 2.8 Statistical analysis

#### 2.8.1 Data analysis and processing

The RevMan 5.3 software was used to conduct statistical analysis. For binary variables, the relative risk ratio (RR) was used for statistics. For continuous variables, standard mean difference (SMD) was selected for statistics; 95% confidence interval (CI) was used to represent the above data, and draw the forest plot. The *I*^2^ test was used to determine the statistical heterogeneity between studies. If *I*^2^ ≤50% and *P* > .1, fixed effect model is adopted to conduct meta-analysis; If *I*^2^ >50% and *P* ≤ .1, random effect model is used to conduct meta-analysis; When there is obvious heterogeneity, subgroup analysis and sensitivity analysis can be conducted to search for heterogeneous sources. If the heterogeneity is large and subgroup analysis is not available, only descriptive analysis is conducted.

#### 2.8.2 Dealing with missing data

If the data of the selected study are missing, we will contact the author by email or phone to obtain the required data. If complete data cannot be obtained, the study is excluded.

#### 2.8.3 Subgroup analysis

Subgroup analysis is conducted according to epicardial adipose tissue measurement threshold. According to the age of the subjects, they are divided into 3 subgroups: youth, middle age, and old age; According to the gender of the subjects, they are divided into 2 subgroups of male and female; Subgroup analysis is conducted according to the severity of coronary artery stenosis; Subgroup analysis is performed according to the diagnostic methods of coronary artery disease.

#### 2.8.4 Sensitivity analysis

The sensitivity analysis is conducted to explore the impact of single study on the merger results to verify the effectiveness of the review conclusion. After eliminating the research with high heterogeneity, repeat the analysis.

#### 2.8.5 Assessment of reporting biases

Funnel plot is used to detect publication bias. If the image is asymmetric, it indicates publication bias. If the number of included studies is more than or equal to 10, Egger and Begg tests can be used.

## 3 Discussion

With the development and change of social life and the increasing of the incidence rate of cardiovascular diseases, cardiovascular disease death is the first cause of death in Chinese residents at present.^[8]^ Early diagnosis and timely intervention are very important for the treatment and prognosis of cardiovascular disease. A large number of studies have shown that epicardial adipose tissue volume and thickness are positively correlated with the occurrence of coronary artery disease.^[9]^ The volume and thickness of epicardial adipose tissue may be taken as the predictive factor of coronary artery disease risk.^[10]^^.^

Under normal conditions, epicardial adipose tissue can play a protective regulation role.^[11]^ However, under the condition of pathological changes, the increase of adipose tissue leads to harmful lipotoxicity and inflammatory factors,^[12]^ and the cytokines or hormones secreted by them have effects on hemodynamics and immunity.^[13]^ The inflammatory factors promote to form the arteriosclerosis,^[14]^ induce the impairment of vascular function,^[15]^ reduce the biological activity of oxidative phosphatase, and cause the organic and functional damage of cardiovascular system.^[16]^

Through this study, we can draw the following conclusions: epicardial adipose tissue is closely related to coronary artery disease.^[17,18]^ However, this study also has some limitations: individual differences of patients are difficult to control, and the results may not be applied to the general population; There are differences in measurement methods and data errors, which may lead to the potential clinical and methodological heterogeneity; This study is a case--control study, which cannot explain the mechanism of epicardial tissue effect on coronary artery disease. At the same time, because only English and Chinese literatures were searched, there may be the publication bias.

## Author contributions

**Data collection:** Baohua Wu and Zhuanqin Ren.

**Data curation:** Baohua Wu, Zhuanqin Ren.

**Funding support:** Bin Hou.

**Literature retrieval:** Baohua Wu and Zhuanqin Ren.

**Software operating:** Zhengang Du and Lei Zhang.

**Supervision:** Lei Zhang.

**Writing – original draft:** Zhuanqin Ren and Zhengang Du.

**Writing – review & editing:** Zhuanqin Ren, Zhengang Du and Bing Hou.
